# Spectral-Domain Measurement of Strain Sensitivity of a Two-Mode Birefringent Side-Hole Fiber

**DOI:** 10.3390/s120912070

**Published:** 2012-09-03

**Authors:** Petr Hlubina, Jacek Olszewski, Tadeusz Martynkien, Pawel Mergo, Mariusz Makara, Krzysztof Poturaj, Waclaw Urbańczyk

**Affiliations:** 1 Department of Physics, Technical University Ostrava, 17. listopadu 15, 708 33 Ostrava-Poruba, Czech Republic; 2 Institute of Physics, Wroclaw University of Technology, Wybrzeże Wyspiańskiego 27, 50-370 Wroclaw, Poland; E-Mails: jacek.olszewski@pwr.wroc.pl (J.O.); tadeusz.martynkien@pwr.wroc.pl (T.M.); waclaw.urbanczyk@pwr.wroc.pl (W.U.); 3 Laboratory of Optical Fibre Technology, Maria Curie-Sklodowska University, Pl. M. Curie-Sklodowskiej 3, 20-031 Lublin, Poland; E-Mails: pawel.mergo@poczta.umcs.lublin.pl (P.M.); pawel.mergo@poczta.umcs.lublin.pl (M.M.); potkris@hermes.umcs.lublin.pl (K.P.)

**Keywords:** microstructured fibers, polarization-maintaining fibers, fiber characterization, fiber optics sensors, interferometry

## Abstract

The strain sensitivity of a two-mode birefringent side-hole fiber is measured in the spectral domain. In a simple experimental setup comprising a broadband source, a polarizer, a two-mode birefringent side-hole fiber under varied elongations, an analyzer and a compact spectrometer, the spectral interferograms are resolved. These are characterized by the equalization wavelength at which spectral interference fringes have the highest visibility (the largest period) due to the zero group optical path difference between the fundamental, the LP_01_ mode and the higher-order, the LP_11_ mode. The spectral interferograms with the equalization wavelength are processed to retrieve the phase as a function of the wavelength. From the retrieved phase functions corresponding to different elongations of a two-mode birefringent side-hole fiber under test, the spectral strain sensitivity is obtained. Using this approach, the intermodal spectral strain sensitivity was measured for both *x* and *y* polarizations. Moreover, the spectral polarimetric sensitivity to strain was measured for the fundamental mode when a birefringent delay line was used in tandem with the fiber. Its spectral dependence was also compared with that obtained from a shift of the spectral interferograms not including the equalization wavelength, and good agreement was confirmed.

## 1. Introduction

A large number of fiber-optic sensor configurations utilizing interference between two spatial or polarization modes are available. Elliptical-core dual-mode fibers that support two stable spatial modes, *i.e.*, the fundamental LP_01_ mode and the first order LP_11_ mode, have been successfully applied to the interferometric sensing of a variety of variables such as strain and temperature [1,2]. The interferometric systems are available even when the group optical path difference (OPD) between two modes is larger than the source coherence length. In this case a tandem configuration of two fiber interferometers, receiving one and a two-mode fiber interferometer, can be used [3,4]. The phase change to be measured is contained in a variation of the far-field modal interference pattern at the sensing fiber end. The other configurations are working in the spectral domain and the phase change to be measured is inscribed in the spectral interference fringes detected by a spectrometer [5,6]. Some of these configurations have been primarily used for measuring the intermodal dispersion in elliptical-core [7] or bow-tie [8] optical fibers and the dispersion of birefringence in elliptical-core optical fibers [9].

Recently, new types of optical fibers, namely microstructured optical fibers, have emerged as active and passive elements in fiber-optic polarimetric and interferometric sensors [10–12]. Most recently, optical fiber sensors based on intermodal interference between core and cladding modes have been proposed and tested for sensing various physical quantities [13–16]. Such interferometric sensors have several advantages over other sensing configurations, including small size, high sensitivity and resolution, fast response time, and low cost. Different configurations to obtain all-fiber intermodal interference in microstructured fibers are reported in literature. One of them is to splice a short piece of the microstructured fiber between two pieces of the same fiber [13] or standard single mode fiber [14]. The other possibility is to make a tapered zone [15], collapse the holes of the microstructured optical fiber at two different places [14,16] or to splice two pieces of microstructured optical fibers with a small offset [16]. In the last approach, different higher order modes can be excited by tuning the lateral offset of the spliced fibers. Most recently, a compact in-line fiber interferometric sensor fabricated in a boron doped two-mode highly birefringent microstructured fiber has been presented [17], which allows for simultaneous measurements of strain and temperature by interrogation of the visibility and the displacement of spectral interference fringes.

In this contribution, the strain sensitivity of a two-mode birefringent side-hole fiber is measured in the spectral domain. A simple experimental setup, comprising a broadband source, a polarizer, a two-mode birefringent side-hole fiber under varied elongations, an analyzer and a compact spectrometer, was used to resolve spectral interferograms. These are characterized by a specific wavelength, the equalization wavelength, at which spectral interference fringes have the highest visibility (the largest period) due to the zero group optical path difference (OPD) between the fundamental, the LP_01_ mode and the higher-order, the LP_11_ mode. The spectral interferograms with the equalization wavelength were processed to retrieve the phase as a function of the wavelength. From the retrieved phase functions corresponding to different elongations of a two-mode birefringent side-hole fiber under test, the spectral strain sensitivity was obtained. To our knowledge, this is the first time the approach is used. Thus, the intermodal spectral strain sensitivity was measured for two orthogonal (*x* and *y*) polarizations. Moreover, the spectral polarimetric sensitivity to strain was measured for the fundamental mode when a birefringent delay line was used in tandem with the fiber. The measured quantity was also compared with that obtained from a shift of the spectral interferograms not including the equalization wavelength (a suitable birefringent delay line was used), and good agreement was confirmed.

## 2. Experimental Method

### 2.1. The Spectral Intensity: A Two-Mode Optical Fiber

Consider a two-mode optical fiber of length *z* characterized by the wavelength-dependent OPD between *x*-polarized modes $\Delta_{10}^{x}(z;\lambda )$. The spectral intensity resolved at the output of the two-mode optical fiber by a spectrometer is [18]

(1) $$I^{x}(z;\lambda )=I_{0}^{x}(\lambda )\;\left\{1+{\text{V}}_{A}^{x}(\lambda )V^{x}(z;\lambda )\cos[2\pi /\lambda ]\Delta_{10}^{x}(z;\lambda )]\right\},$$

where $I_{0}^{x}(\lambda )$ is the reference (unmodulated) spectral intensity, ${\text{V}}_{A}^{x}(\lambda )$ is an aperture visibility term and *V^x^*(*z*; *λ*) is the visibility term given by

(2) $$V^{x}(z;\lambda )=\exp\left\{-(\pi^{2}/2){\left[\Delta_{10}^{gx}(z;\lambda )\Delta \lambda_{\text{R}}/\lambda^{2}\right]}^{2}\right\},$$

where Δ*λ*_R_ is the width of the spectrometer response function and $\Delta_{10}^{gx}(z;\lambda )$ is the intermodal group OPD between the modes. The period of the spectral modulation Λ*^x^*(*λ*) for the *x*-polarized modes is given by

(3) $$\Lambda^{x}(\lambda )=\frac{\lambda^{2}}{\left|\Delta_{10}^{gx}(z;\lambda )\right|}.$$

The equalization wavelength $\lambda_{0}^{x}$ is resolved in the recorded spectrum for which the period is infinite, or equivalently the relation $\Delta_{10}^{gx}(z;\lambda_{0}^{x})=0$ is fulfilled.

Similarly, consider a two-mode optical fiber characterized by the wavelength-dependent OPD between *y*-polarized modes $\Delta_{10}^{y}(z;\lambda )$. The spectral intensity resolved at the output of the two-mode optical fiber by a spectrometer is:

(4) $$I^{y}(z;\lambda )=I^{y(0)}(\lambda )\;\left\{1+{\text{V}}_{A}^{y}(\lambda )V^{y}(z;\lambda )\cos[(2\pi /\lambda )\Delta_{10}^{y}(z;\lambda )]\right\}.$$

The period of the spectral modulation Λ*^y^*(*λ*) for the *y*-polarized modes is given by

(5) $$\Lambda^{y}(\lambda )=\frac{\lambda^{2}}{\left|\Delta_{10}^{gy}(z;\lambda )\right|},$$

where $\Delta_{10}^{gy}(z;\lambda )$ is the intermodal group OPD between the modes. The equalization wavelength $\lambda_{0}^{y}$ is resolved in the recorded spectrum for which the relation $\Delta_{10}^{gy}(z;\lambda_{0}^{y})=0$ is fulfilled.

### 2.2. The Spectral Intensity: Birefringence of the Fundamental Mode

**Figure 1.**
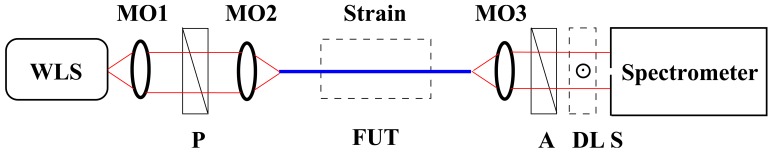
Experimental setup with a fiber under test (FUT) to measure the sensitivity to strain with the remaining components: a white-light source (WLS), microscope objectives 1–3 (MO1, MO2, MO3), a polarizer (P), a fiber under test (FUT), an analyzer (A), a delay line (DL), a spectrometer slit (S).

Consider a linearly polarized optical field, propagating in the positive *z* direction along the axis of a lossless optical fiber (see Figure 1), in which only the fundamental mode in both *x* and *y* polarizations is excited. If the wavelength-dependent phase effective (refractive) indices of the fundamental mode are denoted as *n_x_*(*λ*) and *n_y_*(*λ*), we can define the phase modal birefringence

(6) $$B(\lambda )=n_{x}(\lambda )-n_{y}(\lambda ).$$

Furthermore, using the relation for the group effective (refractive) index

(7) $$N(\lambda )=n(\lambda )-\lambda \frac{\text{d}n(\lambda )}{\text{d}\lambda }=-\lambda^{2}\frac{\text{d}[n(\lambda )/\lambda ]}{\text{d}\lambda },$$

we can express the group modal birefringence *G*(*λ*)

(8) $$G(\lambda )=N_{x}(\lambda )-N_{y}(\lambda )=-\lambda^{2}\frac{\text{d}[B(\lambda )/\lambda ]}{\text{d}\lambda }.$$

The spectral intensity at the output of the configuration shown in Figure 1 with a polarizer and an analyzer oriented 45° with respect to the fiber eigenaxes is [6]

(9) $$I(z;\lambda )=I_{0}(\lambda )\;\left\{1+V(z;\lambda )\cos[(2\pi /\lambda )B(\lambda )z]\right\},$$

where *I*_0_(*λ*) is the reference spectral intensity and the visibility term *V* (*z*; *λ*) is given by

(10) $$V(z;\lambda )=\exp\left\{-(\pi^{2}/2){\left[G(\lambda )z\Delta \lambda_{\text{R}}/\lambda^{2}\right]}^{2}\right\}.$$

The period of the spectral modulation Λ(*λ*) is given by

(11) $$\Lambda (\lambda )=\frac{\lambda^{2}}{\left|G(\lambda )z\right|}.$$

If the resolving power of the spectrometer is insufficient to resolve spectral interference fringes, the fiber in tandem with a birefringent crystal of the phase *B*_c_(*λ*) and group *G*_c_(*λ*) birefringences can be used [6]. The spectral intensity at the output of the tandem configuration shown in Figure 1 with a polarizer and an analyzer oriented 45° with respect to the fiber eigenaxes is [6]:

(12) $$I(z;\lambda )=I_{0}(\lambda )\;\left\{1+V(z;\lambda )\cos\left\{\left(2\pi /\lambda )[B_{\text{c}}(\lambda )d-B(\lambda )z\right]\right\}\right\},$$

where the visibility term *V* (*z*; *λ*) is given by

(13) $$V(z;\lambda )=\exp\{-(\pi^{2}/2)\;{\left\{[G_{\text{c}}(\lambda )d-G(\lambda )z]\Delta \lambda_{\text{R}}/\lambda^{2}\right\}}^{2}\}.$$

The period of the spectral modulation Λ(*λ*) is given by

(14) $$\Lambda (\lambda )=\frac{\lambda^{2}}{\left|G_{\text{c}}(\lambda )d-G(\lambda )z\right|},$$

The equalization wavelength *λ*_0_ is resolved in the recorded spectrum for which the relation *G*_c_(*λ*_0_)*d* = *G*(*λ*_0_)*z* is fulfilled.

## 3. Experimental Configuration

The experimental setup we used to measure the spectral sensitivities to strain is shown in Figure 1. It consists of a white-light source: a supercontinuum source (NKT Photonics), the first microscope objective MO1 (10 × /0.30), polarizer P (LPVIS050, Thorlabs), the second microscope objective MO2 (10 × /0.30), fiber under test FUT, the third microscope objective MO3 (10 × /0.30), analyzer A (LPVIS050, Thorlabs), delay line DL, a spectrometer (USB4000, Ocean Optics) with 25 *μ*m wide slit S and a personal computer. To record the spectral intensity *I^x^*(*z*; *λ*) or *I^y^*(*z*; *λ*), the polarizer and analyzer are oriented 0° or 90° with respect to the major fiber eigenaxis. Similarly, to record the spectral intensity *I*(*z*; *λ*), the polarizer and analyzer are oriented 45° with respect to the fiber eigenaxes and the birefringent delay line (see Figure 1) made of quartz of a suitable thickness with the optical axis oriented 0° with respect to the major fiber eigenaxis is used. Even if many components employed in the experimental setup are bulky and good alignment is necessary, the stability of the measurements is satisfactory.

**Figure 2.**
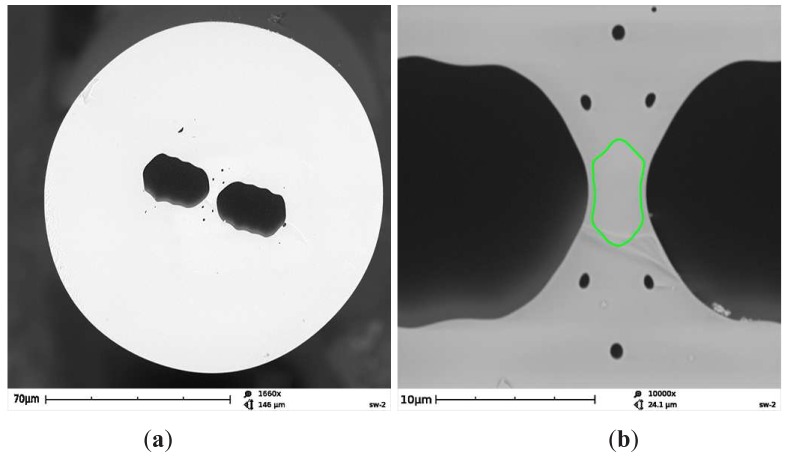
Structure of the fiber under test: general view of the fiber cross-section (**a**), enlarged image of the central region with green line showing core boundary (**b**).

The spectral resolution of the fiber-optic spectrometer USB4000 is in our case given by the slit width. Spectrometer sensitivity is at given light conditions affected by the spectrometer integration time: it can easily be varied under software control and we adjusted it to be 100 ms. The fiber under test is a birefringent side-hole fiber with additional microstructure near the core region composed of six small holes of diameter of about 0.7 *μ*m, which prevent the guided mode to leak out from the core. The fiber was drawn at the Department of Optical Fibers Technology, University of Marie Curie-Sklodowska in Lublin, Poland. As it is shown in Figure 2, the fiber core has elliptical shape of dimensions 6.5 *μ*m × 2.8 *μ*m, while the cladding diameter is 129 *μ*m. The longer axis of the core ellipse is perpendicular to the symmetry axis connecting the centers of the large holes. The initial GeO_2_ concentration in the rod used for preform fabrication was 12 mol%, which corresponds to the refractive index contrast of 0.017 at a 633 nm. However, due to material flow and GeO_2_ diffusion during the fiber drawing process, it is expected that final GeO_2_ concentration in the doped region of the fiber core is much lower. As the glass bridges between the large holes and the doped region of the core are very narrow (about 0.4–0.5 *μ*m) it results in the cutoff of the fundamental mode at about 800 nm.

## 4. Experimental Results and Discussion

### 4.1. Measurement of the Spectral Intermodal Sensitivity to Strain

Using the experimental setup shown in Figure 1, we measured the spectral intermodal sensitivities $K_{10∊}^{x}(\lambda )$ and $K_{10∊}^{y}(\lambda )$ for *x* and *y* polarizations of the modes of the investigated fiber to strain. The parameters are defined by the following relations

(15) $$K_{10∊}^{x}(\lambda )=\frac{1}{L}\frac{\text{d}[\Delta \phi_{10∊}^{x}(\lambda )]}{\text{d}∊},$$

and

(16) $$K_{10∊}^{y}(\lambda )=\frac{1}{L}\frac{\text{d}[\Delta \phi_{10∊}^{y}(\lambda )]}{\text{d}∊},$$

and Equation (15) represents an increase in the phase shift between the two modes in *x* polarization induced by the unit change of strain *∊* acting on unit fiber length. To determine the spectral intermodal sensitivity $K_{10∊}^{x}(\lambda )$ for *x* polarization of the modes of the investigated fiber to strain, we recorded a sequence of spectral interferograms for increasing strain *∊* with a step small enough to assure unambiguity in retrieving the strain-induced phase changes $\Delta [\Delta \phi_{10∊}^{x}(\lambda )]$. To measure the parameter, the fiber of length *L* = 0.895 m was attached with epoxy glue to two translation stages and elongated up to 800 *μ*m (stretched up to 894 *μ*strain). The measurements were repeated several times for increasing and decreasing strain with no hysteresis observed.

**Figure 3.**
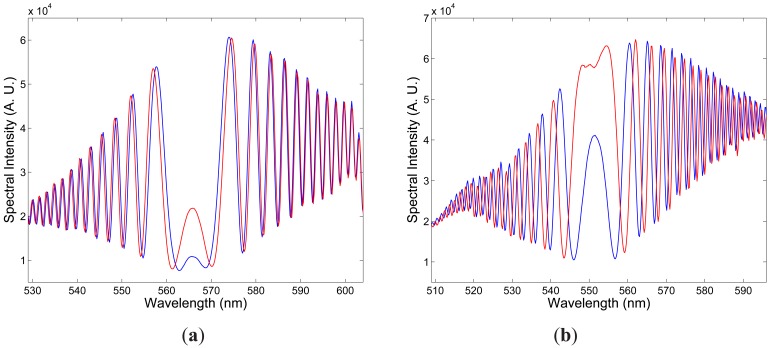
Two examples of the recorded spectra corresponding to two elongations Δ*L*_1_ = 100 *μ*m (blue) and Δ*L*_2_ = 300 *μ*m (red) of the fiber: *x*-polarized modes (**a**), *y*-polarized modes (**b**).

**Figure 4.**
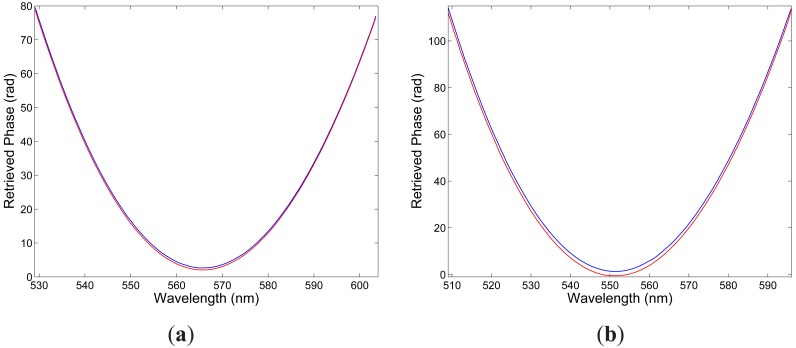
Retrieved phases from the recorded spectra shown in Figure 5 and corresponding to two elongations Δ*L*_1_ = 100 *μ*m (blue) and Δ*L*_2_ = 300 *μ*m (red) of the fiber: *x*-polarized modes (**a**), *y*-polarized modes (**b**).

**Figure 5.**
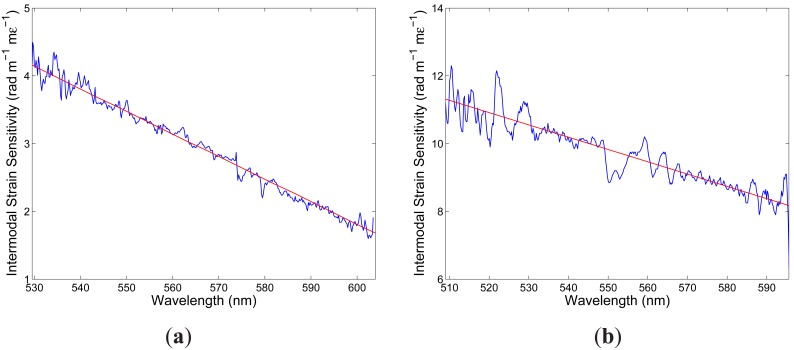
The spectral intermodal sensitivity to strain: *x*-polarized modes (**a**), *y*-polarized modes (**b**). The red lines are the approximate linear dependences obtained from eight measurements.

Figure 3(a) shows two examples of the recorded spectra obtained for *x* polarization of the modes and corresponding to two elongations Δ*L*_1_ = 100 *μ*m and Δ*L*_2_ = 300 *μ*m of the fiber when the overall length of the fiber, including the leading part with length *z*_1_ = 0.565 m, the sensing part with length *L* = 0.895 m and the outer part with length *z*_2_ = 0.835 m, was *z* = 2.295 m. It is clearly seen from the figure that the intermodal interference shows up as the spectral modulation with the wavelength-dependent period and the equalization wavelength $\lambda_{0}^{x}=565.89\;\text{nm}$. The spectral interference fringes for the two elongations are with the same equalization wavelength but with different phases. Using the procedure presented in a previous paper [19], we retrieved from the two spectral interferograms the phase functions $\Delta \phi_{10∊}^{x}(\lambda )$ that are shown in Figure 4(a). These phase functions are wavelength dependent with a minimum at the equalization wavelength $\lambda_{0}^{x}$. From the two phase functions we retrieved the strain-induced phase change $\Delta [\Delta \phi_{10∊}^{x}(\lambda )]$ and the spectral intermodal strain sensitivity $K_{10∊}^{x}(\lambda )$ defined by Equation (15). Figure 5(a) shows by the blue curve the absolute value of $K_{10∊}^{x}(\lambda )$ as a function of wavelength. Figure 5(a) also shows by the red line the approximate linear dependence obtained from eight phase differences retrieved from eight recorded spectral interferograms. It is clearly seen from the figure that the absolute value of the spectral intermodal strain sensitivity $K_{10∊}^{x}(\lambda )$ decreases with a wavelength (in a range of 530 to 600 nm) from a value of 4.2 rad· m^−1^· m*∊*^−1^ to a value of 1.7 rad· m^−1^· m*∊*^−1^.

Similarly, Figure 3(b) shows two examples of the recorded spectra obtained for *y* polarization of the modes and corresponding to two elongations Δ*L*_1_ = 100 *μ*m and Δ*L*_2_ = 300 *μ*m of the fiber. It is clearly seen from the figure that the intermodal interference shows up as the spectral modulation with the wavelength-dependent period and the equalization wavelength $\lambda_{0}^{x}=551.51\;\text{nm}$, which is shifted to the shorter wavelengths in comparison with that for *x*-polarized modes. The spectral interference fringes for the two elongations are with the same equalization wavelength but with different phases. We retrieved from the two spectral interferograms the phase functions that are shown in Figure 4(b). Figure 5(b) then shows by the blue curve the absolute value of the spectral intermodal strain sensitivity $K_{10∊}^{y}(\lambda )$ as a function of wavelength. From the phase functions retrieved from all the recorded spectral interferograms the spectral intermodal sensitivities $K_{10∊}^{y}(\lambda )$ were obtained. Figure 5(b) shows by the red line the approximate linear dependence obtained from eight retrieved phase differences. It is clearly seen from the figure that the absolute value of the spectral intermodal strain sensitivity $K_{10∊}^{y}(\lambda )$ decreases with a wavelength and that it is approximately three time higher and with a greater slope than the spectral intermodal strain sensitivity $K_{10∊}^{x}(\lambda )$.

It is clear from the experimental results that an implementation of a new sensor configuration is possible. In the experimental setup shown in Figure 1 the spectral intensity can be measured for different elongations and from the corresponding recorded spectra the phases can be retrieved. As an example, the retrieved phase can be with the elongation sensitivities similar to those shown in Figure 4(a,b).

### 4.2. Measurement of Spectral Polarimetric Sensitivity to Strain

Using the experimental setup shown in Figure 1 and including delay line DL, we measured the spectral polarimetric sensitivity *K_∊_*(*λ*) of the fundamental mode of the investigated fiber to strain. The parameter is defined by the following relation

(17) $$K_{∊}(\lambda )=\frac{1}{L}\frac{\text{d}[\phi_{x}(\lambda )-\phi_{y}(\lambda )]}{\text{d}∊},$$

and represents an increase in the phase shift between the two polarizations of the fundamental mode, *i.e.*, between the polarization modes, induced by the unit change of strain *∊* acting on unit fiber length [10].

**Figure 6.**
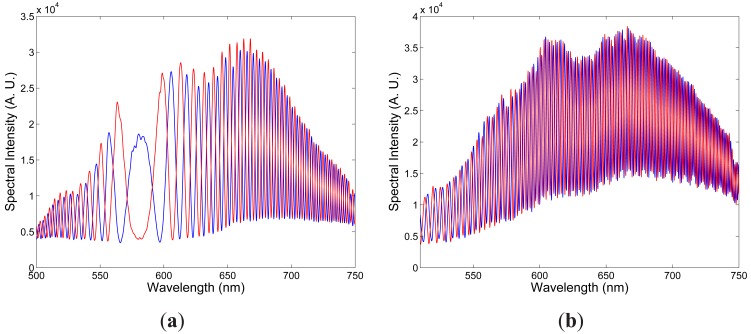
Two examples of the recorded spectra corresponding to two elongations Δ*L*_1_ = 100 *μ*m (blue) and Δ*L*_2_ = 300 *μ*m (red) of the fiber: first delay (**a**), second delay (**b**).

**Figure 7.**
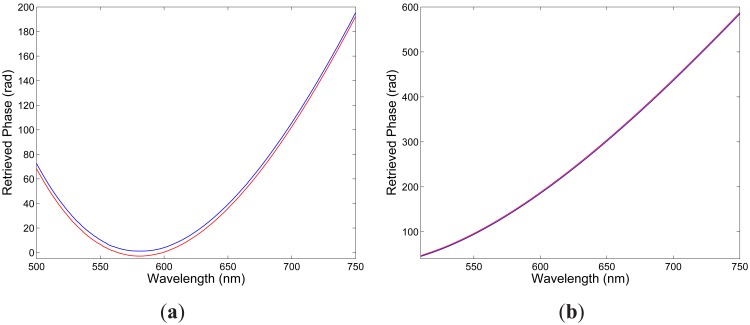
Retrieved phases from the recorded spectra shown in Figure 6 and corresponding to two elongations Δ*L*_1_ = 100 *μ*m (blue) and Δ*L*_2_ = 300 *μ*m (red) of the fiber: first delay (**a**), second delay (**b**).

**Figure 8.**
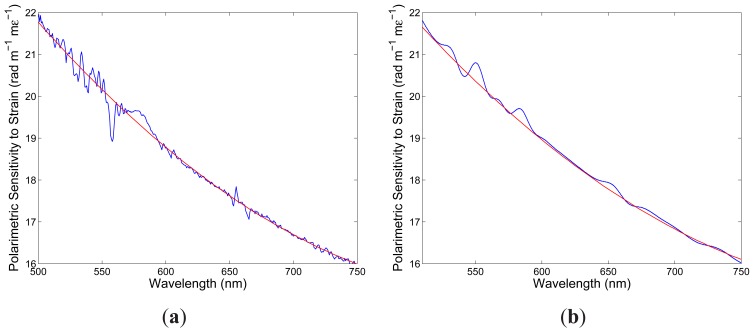
The spectral polarimetric sensitivity to strain: first delay (**a**), second delay (**b**). The red lines are the approximate quadratic dependences obtained from eight measurements.

Figure 6(a) shows two examples of the recorded spectra obtained for a suitable chosen delay line and corresponding to two elongations Δ*L*_1_ = 100 *μ*m and Δ*L*_2_ = 300 *μ*m of the fiber when the overall length of the fiber, including also the sensing part with length *L* = 0.895 m, was *z* = 2.295 m. It is clearly seen from the figure that the interference of polarization modes in the tandem with the delay line shows up as the spectral modulation with the wavelength-dependent period and the equalization wavelength *λ*_0_ = 581.61 nm. The spectral interference fringes for the two elongations are with the same equalization wavelength but with different phases. We retrieved from the two spectral interferograms the phase functions that are shown in Figure 7(a). These phase functions are wavelength dependent with a minimum at the equalization wavelength *λ*_0_. From the phase functions retrieved from eight recorded spectral interferograms the spectral polarimetric sensitivity to strain *K_∊_*(*λ*) for the fundamental mode was obtained. Figure 8(a) shows by the blue curve the absolute value of *K_∊_*(*λ*) as a function of wavelength. Figure 8(a) also shows by the red line the approximate quadratic dependence obtained for eight phase differences retrieved from eight recorded spectral interferograms. It is clearly seen from the figure that the absolute value of the spectral polarimetric sensitivity to strain *K_∊_*(*λ*) decreases with a wavelength (in a range of 500 to 700 nm) from a value of 21.8 rad· m^−1^· m*∊*^−1^ to a value of 16.0 rad· m^−1^· m*∊*^−1^. This sensitivity is approximately three times of that for a birefringent holey fiber [10].

We also measured the spectral polarimetric sensitivity to strain *K_∊_*(*λ*) for the fundamental mode with a different delay line when no equalization wavelength *λ*_0_ is resolvable in the recorded spectrum. In this case the spectral fringes of slightly wavelength-dependent period are resolved in the recorded spectrum and the spectral polarimetric sensitivity to strain *K_∊_*(*λ*) is determined from the change of spectral phase with the elongation of the fiber. As an example, Figure 6(b) shows two examples of the recorded spectra corresponding to two elongations Δ*L*_1_ = 100 *μ*m and Δ*L*_2_ = 300 *μ*m of the fiber. It is clearly seen from the figure that the interference of polarization modes in the tandem with the delay line shows up as the spectral modulation with the wavelength-dependent period and with no equalization wavelength. The spectral interference fringes for the two elongations are with different phases which were retrieved from the two spectral interferograms using a procedure based on the application of a windowed Fourier transform [20]. The corresponding phase functions are shown in Figure 7(b). Figure 8(b) shows by the blue curve the absolute value of the spectral polarimetric sensitivity to strain *K_∊_*(*λ*) for the fundamental mode as a function of wavelength together with the approximate quadratic dependence (red line) obtained for eight phase differences retrieved from eight recorded spectral interferograms. This approach gives the spectral polarimetric sensitivity to strain *K_∊_*(*λ*) with errors (artefacts) smaller in comparison with those obtained by the first approach.

## 5. Conclusions

In this paper, the results of spectral-domain measurements of the strain sensitivity of a two-mode birefringent side-hole fiber have been presented. A simple experimental setup comprising a broadband source, a polarizer, a two-mode birefringent side-hole fiber under varied elongations, an analyzer and a compact has been used to resolve spectral interferograms. These are characterized by the equalization wavelength at which spectral interference fringes have the highest visibility due to the zero group OPD between the fundamental, the LP_01_ mode and the higher-order, the LP_11_ mode. The spectral interferograms with the equalization wavelength were processed by a new method [19] to retrieve the phase as a function of the wavelength. From the retrieved phase functions corresponding to different elongations of the fiber under test, the spectral strain sensitivity was obtained. Using this approach, the intermodal spectral strain sensitivity was measured for two orthogonal (*x* and *y*) polarizations.

Moreover, the spectral polarimetric sensitivity to strain was measured for the fundamental mode when the polarizer and analyzer were oriented 45° with respect to the fiber eigenaxes and a birefringent delay line was used in tandem with the fiber. The measured spectral dependence was also compared with that obtained from a shift of the spectral interferograms not including the equalization wavelength (a suitable birefringent delay line was used), and good agreement was confirmed. The results obtained are important from point of view of implementation of new sensor configurations employing specialty optical fibers such as microstructured side-hole ones.
